# Prediction and prognostic significance of BCAR3 expression in patients with multiple myeloma

**DOI:** 10.1186/s12967-018-1728-8

**Published:** 2018-12-18

**Authors:** Weilong Zhang, Yuansheng Lin, Xiaoni Liu, Xue He, Ye Zhang, Wei Fu, Zuozhen Yang, Ping Yang, Jing Wang, Kai Hu, Xiuru Zhang, Weiyou Liu, Xiaoliang Yuan, Hongmei Jing

**Affiliations:** 1 Department of Hematology, Lymphoma Research Center, Peking University Third Hospital, No. 49 North Garden Road, Haidian District, Beijing, 100191 People’s Republic of China; 2 Gannan Medical University, Ganzhou, 341000 China; 3 Department of Respiratory Medicine, The First Affiliated Hospital of Gannan Medical University, No. 23 Qingnian Road, Zhanggong District, Ganzhou, 341000 People’s Republic of China; 4 Department of Pathology, Beijing Tiantan Hospital Affiliated With Capital Medical University, No. 6 Tiantan Xili, Beijing, 100050 China; 5 Melbourne School of Population and Global Health, The University of Melbourne, Melbourne, VIC 3010 Australia; 6 Peking University Third Hospital, Beijing, 100191 China

**Keywords:** BCAR3, Multiple myeloma, Prognosis, Gene expression profile

## Abstract

**Background:**

Multiple myeloma (MM) is the plasma cell tumor, which is characterized by clonal proliferation of tumor cells, with high risk of progression to renal impairment, bone damage and amyloidosis. Although the survival rate of patients with MM has improved in the past decade, most people inevitably relapse. The treatment and prognosis of MM are still urgent problems. Breast Cancer Antiestrogen Resistance 3 (BCAR3) is a protein-coding gene that is associated with many tumors. However, there have been few studies on the relationship of BCAR3 and MM.

**Methods:**

We analyzed 1878 MM patients (1930 samples) from 7 independent datasets. First, we compared the BCAR3 expression level of MM patients in different stages and MM patients with different amplification of 1q21. Second, we analyzed BCAR3 expression levels in MM patients with different molecular subtypes. Finally, we explored the event-free survival rate (EFS) and overall survival rate (OS) of MM patients with high or low BCAR3 expression, including patients before and after relapse, and their therapeutic responses to bortezomib and dexamethasone.

**Results:**

The expression of BCAR3 showed a decreasing trend in stages I, II and III (P = 0.00068). With the increase of 1q21 amplification level, the expression of BCAR3 decreased (P = 0.022). Patients with high BCAR3 expression had higher EFS and OS (EFS: P < 0.0001, OS: P < 0.0001). The expression of BCAR3 gene before relapse was higher than that after relapse (P = 0.0045). BCAR3 is an independent factor affecting prognosis (EFS: P = 5.17E−03; OS: P = 3.33E−04).

**Conclusion:**

We found that high expression level of BCAR3 predicted better prognosis of MM patients. Low expression of BCAR3 at diagnosis can predict early relapse. BCAR3 is an independent prognostic factor for MM. BCAR3 can be used as a potential biomarker.

**Electronic supplementary material:**

The online version of this article (10.1186/s12967-018-1728-8) contains supplementary material, which is available to authorized users.

## Background

MM is a B cell differentiated tumor characterized by clonal proliferation of tumor cells [1–3]. MM is a heterogeneous disease with different clinical characteristics [4]. By recognizing genetic mechanism and mutation, a normal plasma cell transited into the following disease stages: monoclonal gammopathy of undetermined significance, smouldering myeloma, myeloma and plasma cell leukaemia [5]. The International Staging System (ISS) uses the staging criteria to divide MM into three phases, combining serum albumin levels with β2-microglobulin to determine the prognosis of MM patients [6]. Revised International Staging System (R-ISS) is a simple and effective prognostic staging system that combines three prognostic tools (ISS: International Staging System, CA: chromosomal abnormalities, LDH: lactate dehydrogenase) to better assess patient prognosis [7]. Gene expression profiling (GEP) is important for revealing MM molecular heterogeneity of different patients. The molecular classification established by UAMS (The University of Arkansas for Medical Sciences) based on GEP data [8]. The molecular basis of MM is defined by unsupervised clustering analysis of mRNA expression profiles, which are divided into seven molecular subtypes [9]. Recurrence is a major problem in most MM patients. Among all MM patients, early recurrence and poor prognosis are about 10% to 15% [10].

1q21 amplification is the most common chromosomal aberration in MM and is considered a high-risk genetic feature [11]. The function of the BCAR3 (Breast Cancer Antiestrogen Resistance 3) gene is varied. BCAR3 can promote cell migration, proliferation, and BCAR3 was identified as a molecular linkage between PTP and Cas [12–14]. BCAR3 protein participates in the signaling pathway of EGF through its SH2 domain, leading to cell cycle progression, and BCAR3 itself belong to a mitogenic signaling pathway [15, 16]. BCAR3 and p130Cas were associated with anti-estrogen in breast cancer, Rac activation. BCAR3 can regulate the Src signal transmission of BCAR3-p130 (cas) complex dependence mode [17–19]. During the initial stage of gonadal development, BCAR3 in gonad development is very important and is expressed in XY gonads [20]. Studies have shown that the BCAR3 gene is the first spontaneous mutation associated with cataracts caused by lens compression. This new cataract model could provide further knowledge about the function of BCAR3 protein [21]. In the study of tamoxifen in the treatment of metastatic breast cancer, high expression of BCAR3 is related to good progression-free survival, and the expression level of BCAR3 in primary breast tumors is relatively low, which is related to the survival rate of distant metastasis [14]. Ovarian cancer is a disease characterized by tumor heterogeneity, which is difficult to be diagnosed and treated. Studies have shown that inhibiting the expression of BCAR3 gene can inhibit the cell proliferation of ovarian cancer [22]. However, there is no study reporting the relationship between BCAR3 and MM so far. By integrating data of 1878 MM patients, we found that BCAR3 gene is closely related to MM.

## Methods

### Data source

In our study, gene expression microarrays of 1878 MM patients (1930 samples) were derived from Gene Expression Omnibus database, including datasets GSE24080 (559 samples) [23], GSE82307 (66 samples) [24], GSE19784 (308 samples) [25], GSE83503 (585 samples) [26], GSE9782 (238 samples) [27], GSE39754 (136 samples) and GSE19554 (38 samples) [28, 29]. The criteria for patient selection in our research were stated. 1) All MM patients with the published high throughput gene expression data. 2) All the patients should have some information such as clinical features, biochemical examination, karyotype, therapy or therapy response. The study was approved by the Human Research Ethics Committee of Peking University third hospital. The research was conducted in accordance International Conference on and the Declaration of Helsinki.

### Microarray analysis

All microarray data were analyzed, and the significantly abnormal expression genes were systematically screened as predictive biomarkers. The different expression of genes between BCAR3-low to BCAR3-high group were also analyzed and ranked by foldchange values (log2, FC > 0.8 or < − 0.8, P < 0.05).

We retrieved GSE24080 (559 samples) from the NCBI GEO database and analyzed the expression of BCAR3 among different ISS stages, different molecular subtypes, different 1q21 amplification levels, and different survival (EFS and OS). We retrieved GSE82307 (66 samples) from the NCBI GEO database. We analyzed the expression of BCAR3 before and after recurrence in the same patient. We retrieved GSE19554 (38 samples) in 19 MM patients from the NCBI GEO database, and we analyzed BCAR3 expression between the baseline (before chemotherapy) and pre-1st (after induction of chemotherapy, before bone marrow transplantation) in the same sample. We retrieved GSE19784 (308 samples) from the NCBI GEO database and analyzed the expression of BCAR3 in different molecular subtypes. We retrieved GSE83503 (585 samples) from the NCBI GEO database and analyzed the expression of BCAR3 in the recurrence and non-recurrence groups. We retrieved GSE9782 (238 samples) from the NCBI GEO database and analyzed the expression of BCAR3 between the different treatment response of bortezomib and dexamethasone. All patients from GSE39754 (136 samples) were treated with vincristine, adriamycin, and dexamethasone (VAD), followed by autologous stem cell transplantation (ASCT). Then measuring “therapeutic response” after ASCT. Analysis of BCAR3 expression in each treatment response compared to the average of all treatment responses. Therapeutic response: complete response (CR), very good partial response (VGPR), partial response (PR), no response, stable disease (NR), no response, progressive disease (Prog).

### Gene ontology (GO) analysis

Use the DAVID to analyze the 559 samples (dataset GSE24080), and find out the enrichment pathways for different expressed genes between BCAR3-low and BCAR3-high group [30]. The results were ranked by the P value (− log10, P < 0.05).

### Statistical analyses

Statistical analyses were performed by R software v3.1.3 (ggplot2 and survminer package). The log-rank test and cox regression multivariate analysis were used for survival analysis. P value < 0.05 was considered statistically significant.

## Result

### BCAR3 is expressed in multiple myeloma of different molecular types

**Fig. 1:**
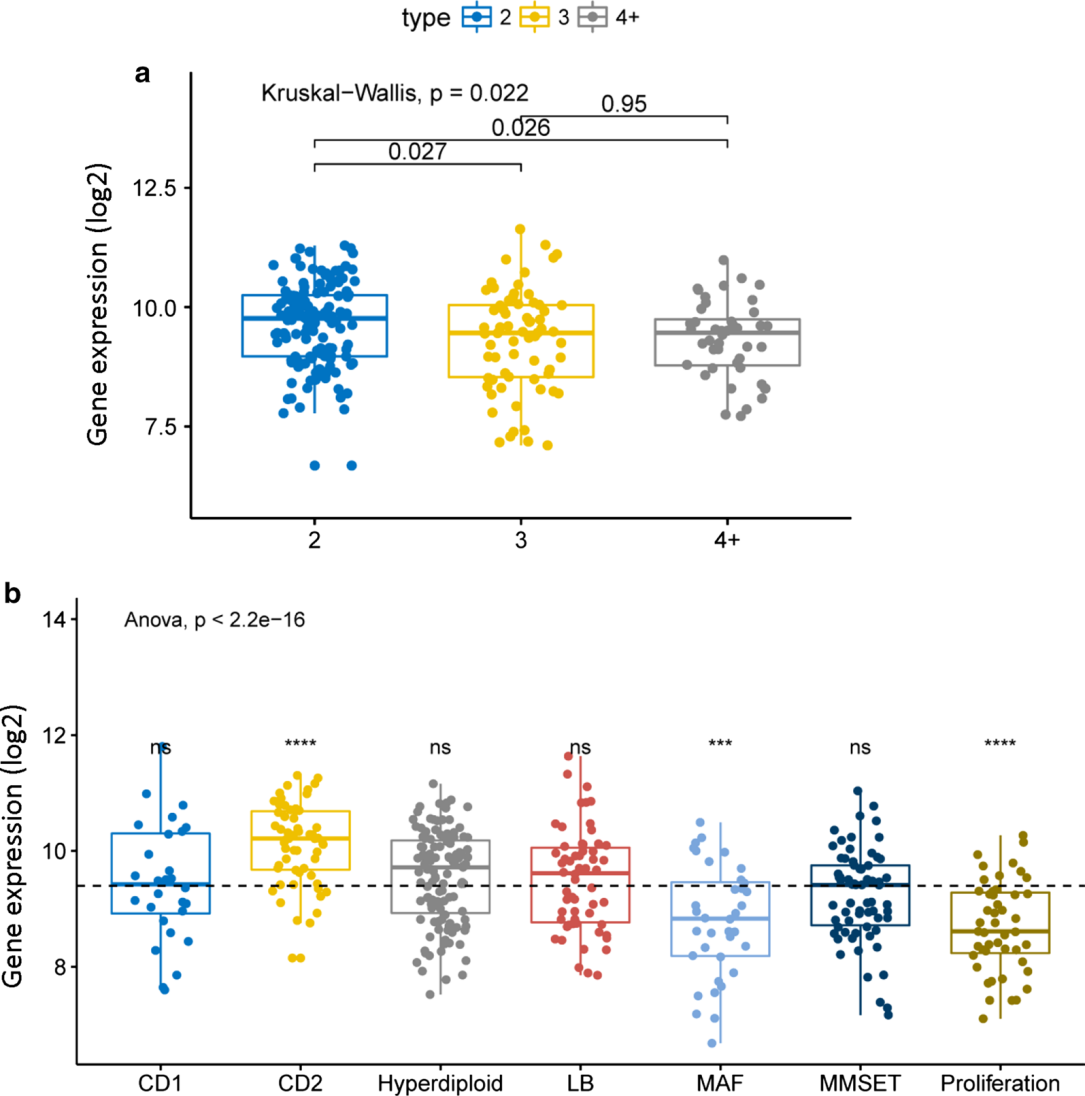
The expression of BCAR3 in different amplification levels of 1q21 and 7 molecular subtypes. **a** BCAR3 expression levels at different amplification levels of 1q21. The X-axis represents the 1q21 amplification, the Y-axis represent BCAR3 expression level. P = 0.022, Kruskal–Wallis test. **b** Comparison of BCAR3 expression levels in 7 molecular subtypes. The Y-axis represents BCAR3 expression levels; the X-axis represents 7 molecular subtypes. P < 2.2E−16, Anova test. We use statistically significant symbol: ns; P > 0.05; *P < = 0.05; *P < = 0.01; * *P < = 0.001; * * * *P < = 0.0001

We compared BCAR3 gene expression level of different 1q21 amplification. With the increase of the amplification level of 1q21, the expression level of BCAR3 showed an overall downward trend (Fig. 1a, P = 0.022, Kruskal–Wallis test). New UAMS classifies MM into seven subtypes based on different gene expression profiles. Comparison of expression levels in seven molecular types showed that the expression level of BCAR3 gene is different in molecular subtypes. CD2 subtype had the highest BCAR3 expression, while the proliferation (PR) subtype and MAF subtype had the lowest BCAR3 expression (Fig. 1b, P < 2.2E−16, Anova test). We also analyzed the dataset GSE19784, a total of 308 samples, the expression level of BCAR3 in CD1 subtype and CD2 subtype was the highest, while that of PR (proliferation) subtype was the lowest (Additional file 1: Figure S1, P = 7.8E−10, Anova test).

### Relapse is associated with low BCAR3 expression

**Fig. 2:**
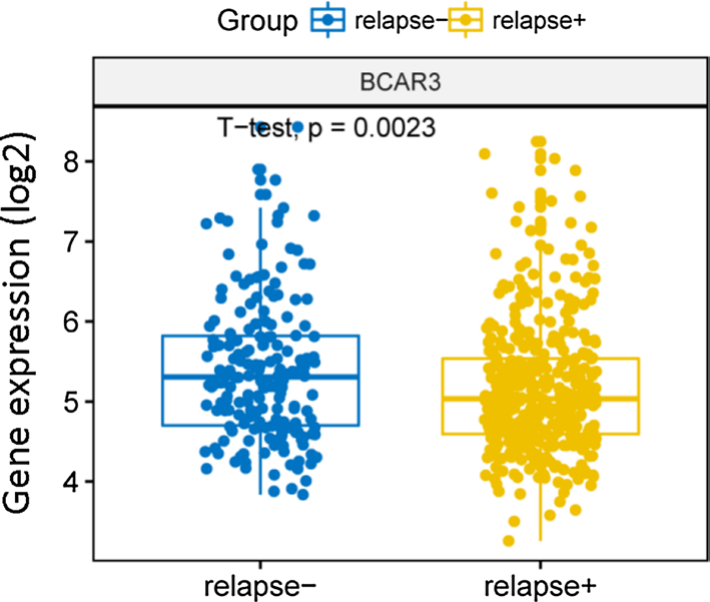
The expression level of BCAR3 in the relapse group and the non-relapse group from dataset GSE83503 (585 samples). The blue one represents non-relapse group and the yellow one represents relapse group. P = 0.0023, Unpaired t test, two sided

The expression of BCAR3 gene was statistically different in the non-relapse group and the relapse group of MM patients in dataset GSE83503 (585 samples), and the expression in the non-relapse group was significantly higher than that in the relapse group (Fig. 2, P = 0.0023, Unpaired t test, two sided). Therefore low expression of BCAR3 at diagnosis can predict early relapse.

**Fig. 3:**
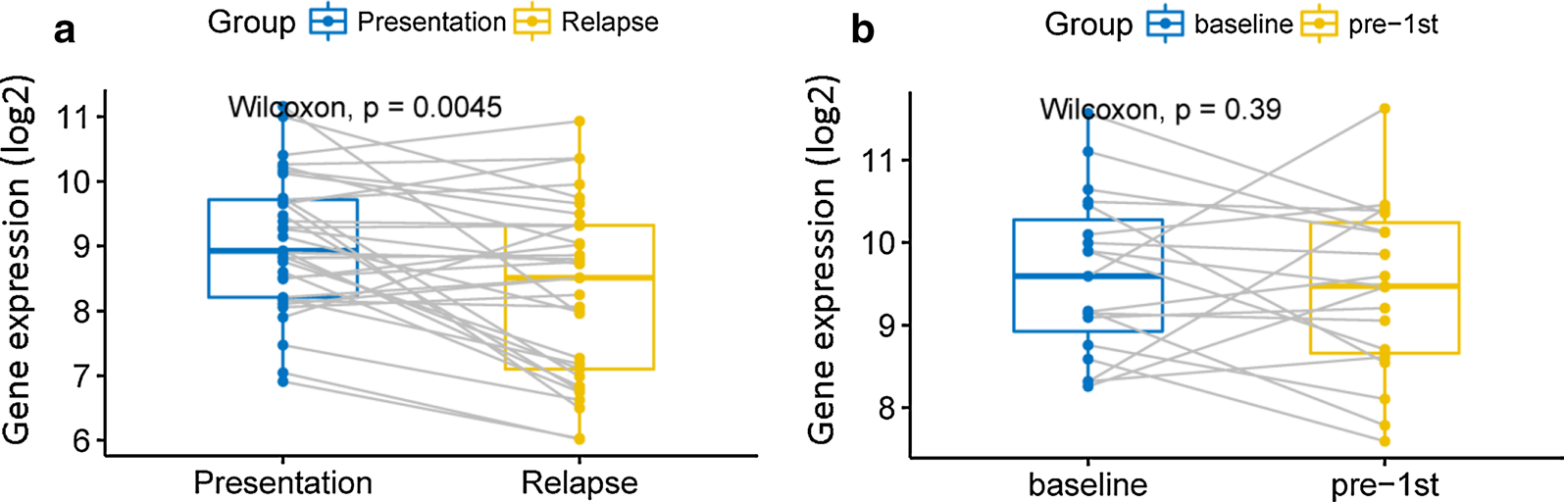
We compared BCAR3 gene expression before and after therapy. Gray lines represent a trend in the same indicator for a same patient. **a** BCAR3 expression levels were compared before and after relapse in the same patient. P = 0.0045, Wilcoxon test. **b** The expression levels of BCAR3 baseline and pre-1st were compared. P = 0.39, Wilcoxon test

The expression of BCAR3 gene before (at diagnosis) and after relapse (in remission) was compared in dataset GSE82307 (66 samples). The expression of BCAR3 gene in the same MM patients before and after relapse was statistically different, and the expression before relapse was significantly higher than that after recurrence (Fig. 3a, P = 0.0045, Wilcoxon test). However, the expression of BCAR3 gene was not changed before and after chemotherapy in the same patient (Fig. 3b, P = 0.39, Wilcoxon test).

### BCAR3 gene can predict survival of MM

**Fig. 4:**
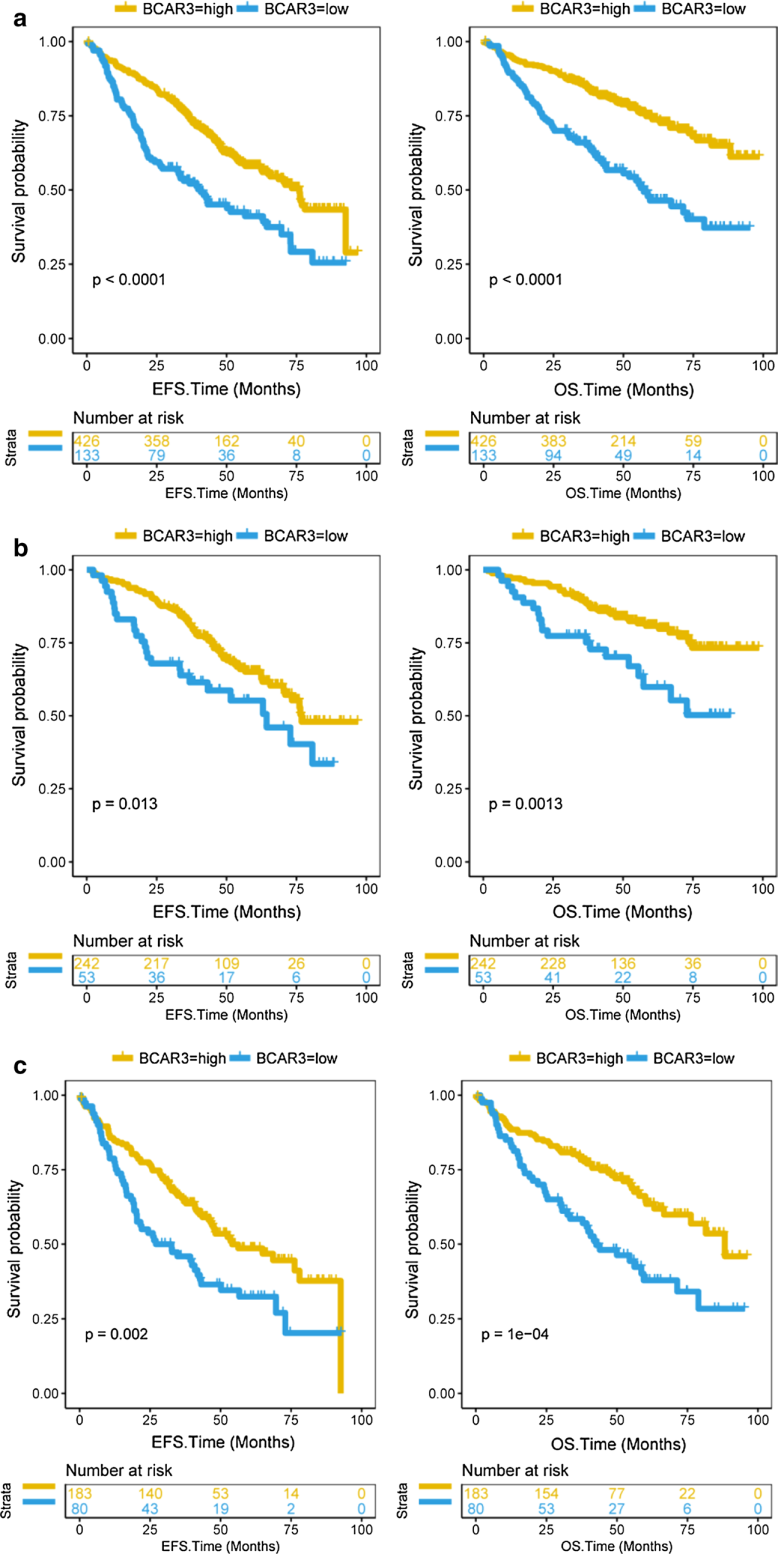
The survival of EFS and OS in BCAR3-low and BCAR3-high group. The X-axis represents the EFS/OS time (months), the Y-axis represents the survival probability. Yellow line represents BCAR3-low group, blue line represents BCAR3-high group. EFS: event-free survival rate; OS: overall survival rate. **a** The survival of EFS and OS in BCAR3-low and BCAR3-high group from 559 patients. EFS: P < 0.0001, OS: P < 0.0001, Log-rank test. **b** Survival curve in patients with ISS stage I. EFS: P = 0.013, OS: P = 0.0013, Log-rank test. **c** Survival curve in patients with ISS stage II or III between BCAR3-high and BCAR3-low group. The X-axis represents the EFS/OS time (months); the Y-axis represents the survival probability. EFS: P = 0.002, OS: P = 1E−04, Log-rank test

Patients with high BCAR3 expression had higher EFS and OS, while low BCAR3 expression had lower EFS and OS in dataset GES24080 (559 samples) (Fig. 4a, EFS: P < 0.0001, OS: P < 0.0001, Log-rank test). In the survival curve of ISS stage I patients, we can see that patients of ISS stage I have higher EFS and OS when BCAR3 gene is highly expressed (Fig. 4b, EFS: P = 0.013, OS: P = 0.0013, Log-rank test). Patients of ISS stage II and III also have higher EFS and OS when BCAR3 gene is highly expressed (Fig. 4c, EFS: P = 0.002, OS: P = 1E−04, Log-rank test).

### BCAR3 higher expressed in stage I than stage II and III in multiple myeloma

The expression levels of BCAR3 in GSE24080 dataset (559 samples) were compared at different ISS stages. The expression of BCAR3 showed a decreasing trend in stages I, II and III (Additional file 1: Figure S2A, P = 0.00068, Kruskal–Wallis test). There was no statistically significant difference between stage II and III (Additional file 1: Figure S2A, P = 0.39, Wilcoxon test). In addition, the expression levels of BCAR3 under different serotype stratification were compared at different stages. In serum immunoglobulin A (IgA) group and serum immunoglobulin G (IgG) group, the expression of BCAR3 in stages I, II and III decreased gradually (Additional file 1: Figure S2B, IgA: P = 0.013; IgG: P= 0.04; Kruskal–Wallis test). However, there was no statistical significance in the serum free light chain (FLC, light chain myeloma subtype) group (Additional file 1: Figure S2B, FLC: P = 0.39; Kruskal–Wallis test).

### The BCAR3 gene is associated with immunity related pathway

68 up-regulated and 23 down-regulated genes were found between the BCAR3-high and BCAR3-low group. Heat map shows top 12 up-regulated genes and top 12 down-regulated genes (Additional file 1: Figure S3A, P < 0.01). Among the enriched pathway of different expressed gene, immune response pathway (GO: 0006955) and B cell receptor signaling pathway (GO: 0050853) are the most related (Additional file 1: Figure S3B, P < 0.01). In the immune response pathway, all the 11 different expressed genes such as CCL18, CD27, CD74, CTSW and CXCL12 were up-regulated in the BCAR3-high group compared with the BCAR3-low group (Additional file 1: Figure S4, P < 0.001, Unpaired t test, two sided).

### Comparison of BCAR3 expression of different therapeutic responses to bortezomib and dexamethasone

BCAR3 expression of different therapeutic responses to bortezomib and dexamethasone was compared in 238 samples of GSE9782 dataset. There was no difference in the expression of the BCAR3 in the post-treatment responses of bortezomib and dexamethasone (Additional file 1: Figure S5, bortezomib: P = 0.21 dexamethasone: P = 0.65, Anova test). There was also no significant difference in the expression of BCAR3 in each treatment response in 136 MM patients from GSE39754 dataset (Additional file 1: Figure S6, P = 0.96, Anova test).

### BCAR3 is an independent factor affecting prognosis of MM

Cox regression multivariate analysis was used to compare the clinical characteristics of 559 patients with MM. In the EFS, B2M (HR = 1.38, 95% CI 1.01–1.88; P = 4.22E−02), MRI (HR = 1.40, 95% CI 1.07–1.84; P = 1.47E−02), BMPC (HR = 1.39, 95% CI 1.01–1.92; P = 4.56E−02) and BCAR3 (HR = 0.66, 95% CI 0.5–0.88; P = 5.17E−03) are prognostic factors for MM. BCAR3 (P = 5.17E−03) is a prognostic factor independent of B2M, MRI, and BMPC in myeloma patients (Additional file 2: table S1). In the OS, B2M (HR = 1.59, 95% CI 1.09–2.33; P = 1.74E−02), MRI (HR = 1.80, 95% CI 1.28–2.52; P = 7.52E−04), and BCAR3 (HR = 0.55, 95% CI 0.39–0.76; P = 3.33E−04) are prognostic factors for MM (Additional file 2: Table S1). BCAR3 is the most statistically significant biomarker of all variable that we analyzed in both EFS and in OS (Additional file 2: Table S1, EFS: P = 5.17E−03, OS: P = 3.33E−04, Cox regression multivariate analysis). BCAR3 is an independent factor in the prognosis of MM.

We also analyzed whether the clinical characteristics were significantly different between BCAR3-low group (133 samples) and BCAR3-high group (426 samples). Age (P = 0.219), sex (P = 0.733), race (P = 0.937) and isotypes (P = 0.414) were not statistically significant (Additional file 2: Table S2, Fisher’s exact test). B2M (P = 0.004), LDH (P = 0.007), HGB (P = 0.038), ASPC (P = 0.031), BMPC (P < 0.001) and MRI (P = 0.001) are significantly different in BCAR3-low group and BCAR3-high group (Additional file 2: Table S2, Unpaired t test, two sided). CRP (P = 0.738), CRECT (P = 0.426) and ALB (P = 0.082) were not significantly different in BCAR3-low group and BCAR3-high group (Additional file 2: Table S2, Unpaired t test, two sided).

## Discussion

For MM patients with age less than 65 years, high-dose treatment with autologous stem cells is a first-line effective treatment [31]. But MM is a kind of incurable disease and a B-cell differentiated tumor, whose character is cloned plasma cell proliferations, kidney failure, anemia, dissolve the bony lesions, hypercalcemia and infection [32–34]. Free monoclonal immunoglobulins in serum or urine were found to be related with malignant plasma cell marrow infiltration [35, 36]. BCAR3 is a protein-coding gene, diseases associated with BCAR3 include estrogen resistance, breast cancer, and cataracts. However, the relationship between BCAR3 and MM has not been studied. Therefore, we analyzed the expression level of BCAR3 gene in patients with MM, and found that high BCAR3 expression has better prognosis.

Different mechanisms for BCAR3 in cancer were reported. (1) The interaction between BCAR3 and cas is blocked by the L744E/R748E mutation of BCAR3, the activity of Rac1 is decreased, and the tension of RhoA may be increased, which provides stable adhesion and slows disassembly, thus inhibiting tumor progression. In contrast, BCAR3 and cas interact to activate Rac1, rapidly breaking down adhesion and resulting tumor invasion [16]. (2) High expression of AND-34/BCAR3 activates the activity of Rac and Pak1, thereby activating the CyclinD1 promoter, making anti-estrogen resistance and progression of breast cancer cells. In contrast, a dominant negative for Rac and Pak1 inhibits the activation of the CyclinD1 promoter [37]. (3) It is believed that breast tumors initially depend on estrogen for development. Through experiments, BCAR3 is closely related to in vitro anti-estrogen resistance. There is an inverse relationship between BCAR3 expression and ER (estrogen receptor) protein expression in breast and ovarian cells. Tumor cell growth inhibition and hormonal signal blockade are due to the competition of ER by antiestrogens. When BCAR3 is low expressed, ER is highly expressed and inhibits tumor progression [38]. (4) TGF-β/Smad is a signaling pathway of BCAR3 in breast invasive tumors, and BCAR3 inhibits the conduction of TGFβ/Smad signaling when highly expressed, thereby inhibiting tumor progression [14]. So, overexpression of BCAR3 in breast cancer cells can promote cell migration and invasion in most researches. However, in some researches, overexpression of BCAR3 in breast cancer cells can inhibit cell migration and invasion. In our research, we found that BCAR3 is closely related to the immune response pathway (Additional file 1: Figure S3B). All the 11 different expressed genes such as CCL18, CD27, CD74, CTSW and CXCL12 were up-regulated in the BCAR3-high group compared with the BCAR3-low group, which suggest that in the immune response pathway was activated (Additional file 1: Figure S4). For example, CXCL12 is a gene in the immune response pathway. The expression of CXCL12 inhibits the metastasis and growth of primary breast cancer [39]. So, high expression of BCAR3 may inhibit MM growth through up-regulated genes in the immune response pathway.

High dose autologous bone marrow transplantation has been reported and achieved good results [40]. Pre-relapse mortality was significantly lower than post-relapse [41]. The survival rate of patients after relapse is reduced. Our study shows that in patients with MM, low expression of BCAR3 at diagnosis can predict early relapse. In addition, MM is a clonal plasmacytoma that can be judged by monoclonal immunoglobulin (IgA, IgG) or increased light chain levels [42]. The heavy/light chain assay can predict the prognosis and surveille disease in patients with myeloma after treatment [43]. We also studied serum IgA and serum IgG as well as light chain and further analyzed the expression of BCAR3 at different stages of each serotype. In serum FLC, there was no significant regularity in the expression of BCAR3 between stages I, II and III. However, in the serum IgA group and the serum IgG group, the expression of BCAR3 of stage I MM was higher than that of stage II and stage III.

BCAR3 is an independent prognostic factor for MM, which can reflect the survival rate of patients with MM. We have some important evidence to support this view: First, patients with high BCAR3 expression had higher EFS and higher OS. Second, MM patients with BCAR3 had higher expression before relapse and lower expression after relapse. Last, B2M, LDH, HGB, ASPC, BMPC and MRI have significant differences in the BCAR3-low and BCAR3-high groups (Additional file 2: Table S2). It was found that high expression of BCAR3 gene predicted good prognosis.

The study has some shortcomings. For example, the molecular mechanism of BCAR3 gene in MM is not in-depth studied. We can also further study from this aspect. In addition, MM may be further divided into low risk, moderate risk and high risk combined the expression level of BCAR3 and other known biomarkers, which is helpful to evaluate the survival of patients. These shortcomings needs to be further studied.

## Conclusions

In summary, our data show that high expression levels of BCAR3 predict a better prognosis for MM patients. Low expression of BCAR3 at diagnosis can predict early relapse. BCAR3 is an independent prognostic factor for MM. BCAR3 can be used as a potential biomarker.

## Additional files

**Additional file 1: Figure S1.** The expression level of BCAR3 in different molecular subtypes. The X-axis represents the 9 molecular subtypes; the Y-axis represents the gene expression. The dotted line represents the average of BCAR3 gene expression levels of all molecular subtypes, including CD1, CD2, CTA, HY, MF, MS, myeloid, NFKB, PR. P = 7.8E−10, Anova test. **Figure S2.** The expression level of BCAR3 in different ISS stages and 3 serotypes in MM patients. A, The expression of BCAR3 was compared between different ISS stages in MM patients. The X-axis represents the ISS stages; the Y-axis represents BCAR3 expression level (log2). P = 0.00068, Kruskal–Wallis test. B, The expression of BCAR3 in different ISS stages were compared under different serotype stratification (FLC: Serum free light chain, IgA: Serum immunoglobulin A, IgG: serum immunoglobulin G). Kruskal–Wallis test. **Figure S3.** Heat map of different expression genes between BCAR3-low and BCAR3-high groups and related enrichment pathways. A, Heat map shows top 12 up-regulated genes and top 12 down-regulated genes. The red represents high expression, the white represents intermediate expression, and the green represents low expression. The foldchange (log2) of different expressed genes is ranked, and the corresponding P-value (− log10) is on the right in the heat map. B, The enrichment pathways for different expression genes. The X-axis represents p-value (− log10) and the Y-axis represents different enriched pathways. **Figure S4.** The expression levels of 11 different genes in the immune response pathway in the BCAR3-high group and the BCAR3-low group were compared. Unpaired t test, two sided. **Figure S5.** BCAR3 expression in different therapeutic response to bortezomib and dexamethasone. The left side shows the therapeutic response to bortezomib. The therapeutic response to dexamethasone was shown on the right. The expressions of BCAR3 were compared between complete remission (CR), partial remission (PR), minimal response (MR), no change (NC), and disease progression (DP) group. The dotted line represents the average of BCAR3 gene expression levels in all treatment responses. Bortezomib: P = 0.21, dexamethasone: P = 0.65, Anova test, two sided. Statistical significance: ns: P > 0.05; *: P < = 0.05; **: P < = 0.01; ***: P < = 0.001; ****: P < = 0.0001. **Figure S6.** Comparison of expression levels of the BCAR3 gene in therapeutic responses. The X-axis represents the groups of treatment responses to induction chemotherapy and autologous stem cell transplantation; the Y-axis represents the expression of BCAR3. The dotted line represents the average of BCAR3 gene expression levels in all treatment responses. Treatment responses: Complete Response (CR); Very Good Partial Response (VGPR); Partial Response (PR); No Response, Stable disease (NR); No Response, Progressive disease (Prog). P = 0.96, Anova test. Statistical significance: ns: P > 0.05 *: P < = 0.05 **: P < = 0.01 ***: P < = 0.001 ****: P < = 0.0001.

**Additional file 2: Table S1.** Multivariate analysis of clinical prognostic parameters in 559 multiple myeloma patients (Cox regression multivariate analysis). **Table S2.** Baseline patient characteristics according to the expression level of BCAR3.

## Abbreviations

MM: multiple myeloma
EFS: event-free survival time, defined from date of registration to the occurrence of death from any cause, disease progression or relapse, or censored at the date of last contact
OS: overall survival time, defined from date of registration to the date of death from any cause or censored at the date of last contact
B2M: beta-2 microglobulin
ALB: albumin
HGB: haemoglobin
MRI: number of magnetic resonance imaging (MRI)-defined focal lesions (skull, spine, pelvis)
BMPC: bone marrow biopsy plasma cells (%)
HR: hazard ratio
CI: confidence interval
CRP: C-reactive protein
CREAT: creatinine
ASPC: aspirate plasma cells (%)
