# Supplementary material for: Prediction and prognostic significance of BCAR3 expression in patients with multiple myeloma
Source: J Transl Med. 2018 Dec 18;16:363. doi: 10.1186/s12967-018-1728-8 (PMC6299524; doi:10.1186/s12967-018-1728-8)

Additional Figure S1

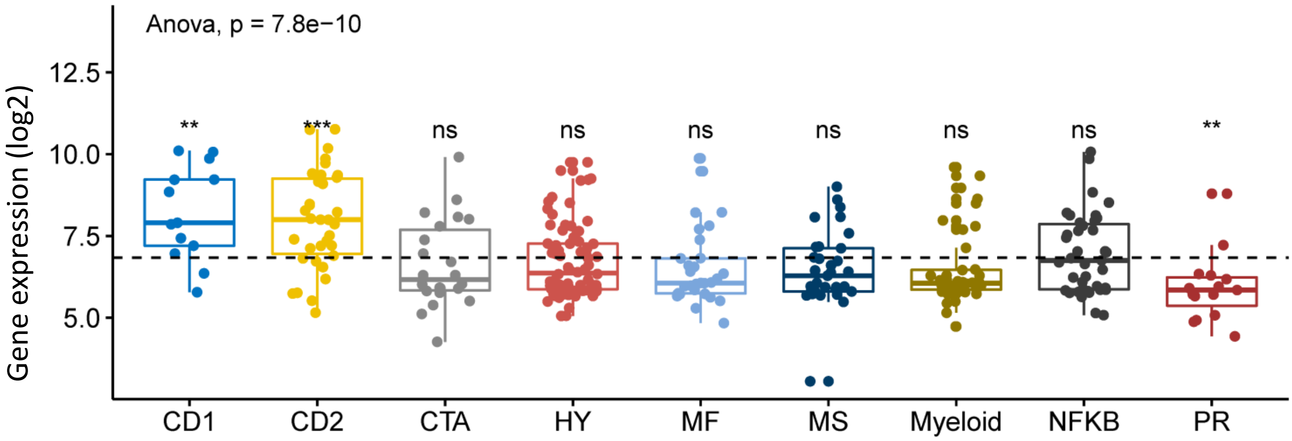

Additional Figure S2

A

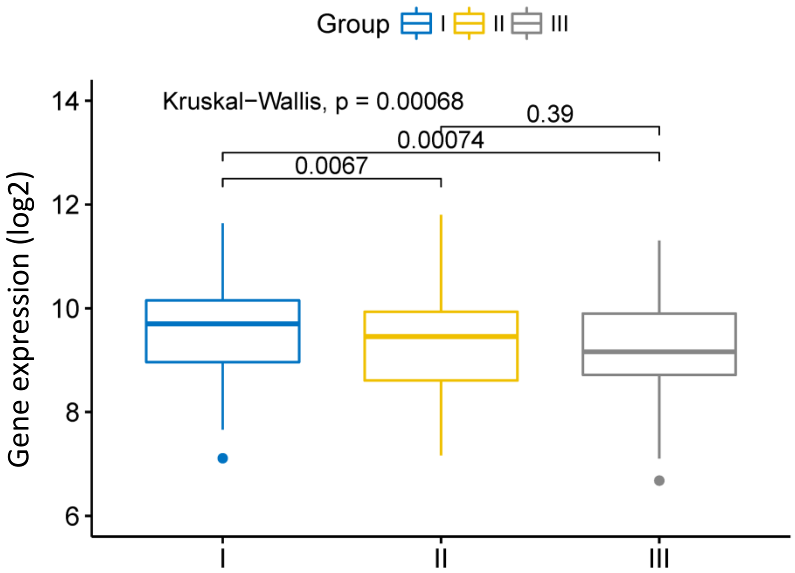

B

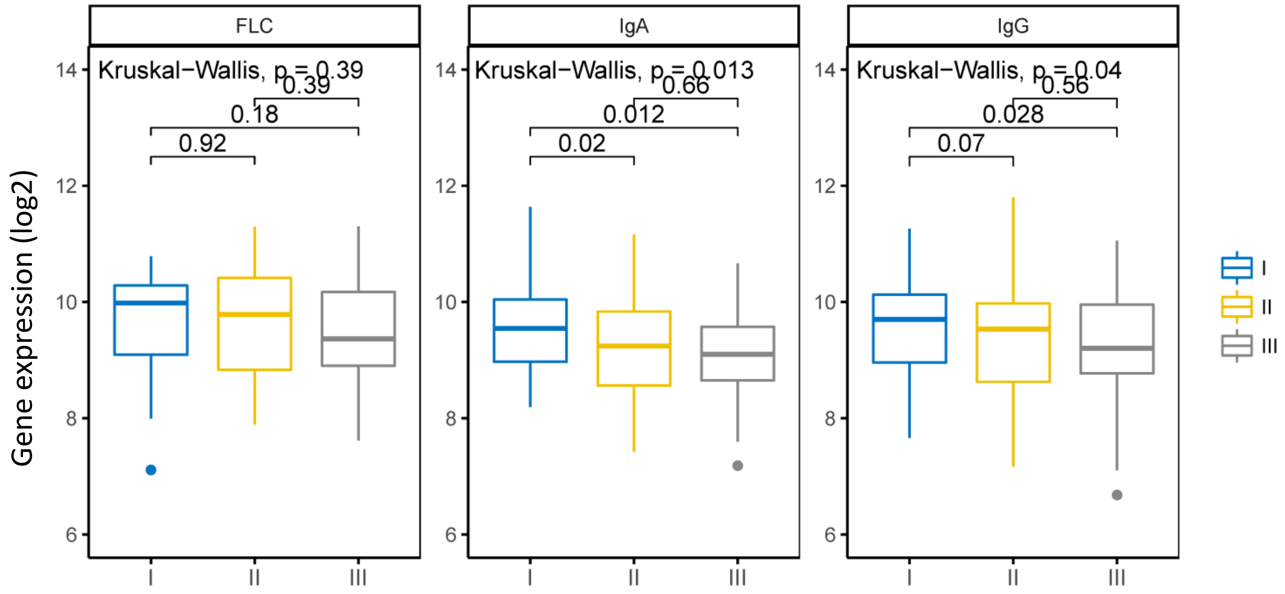

Additional Figure S3

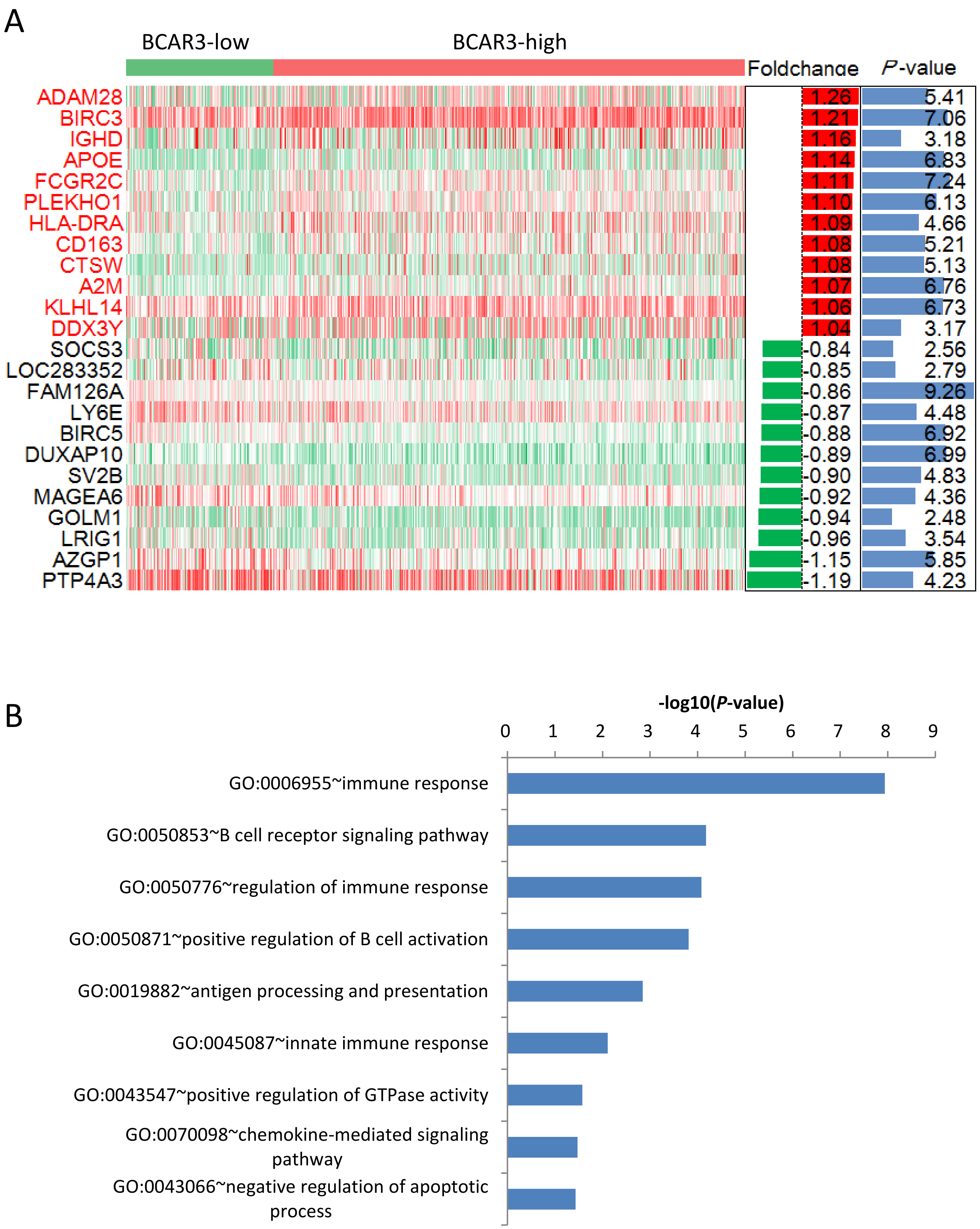

Additional Figure S4

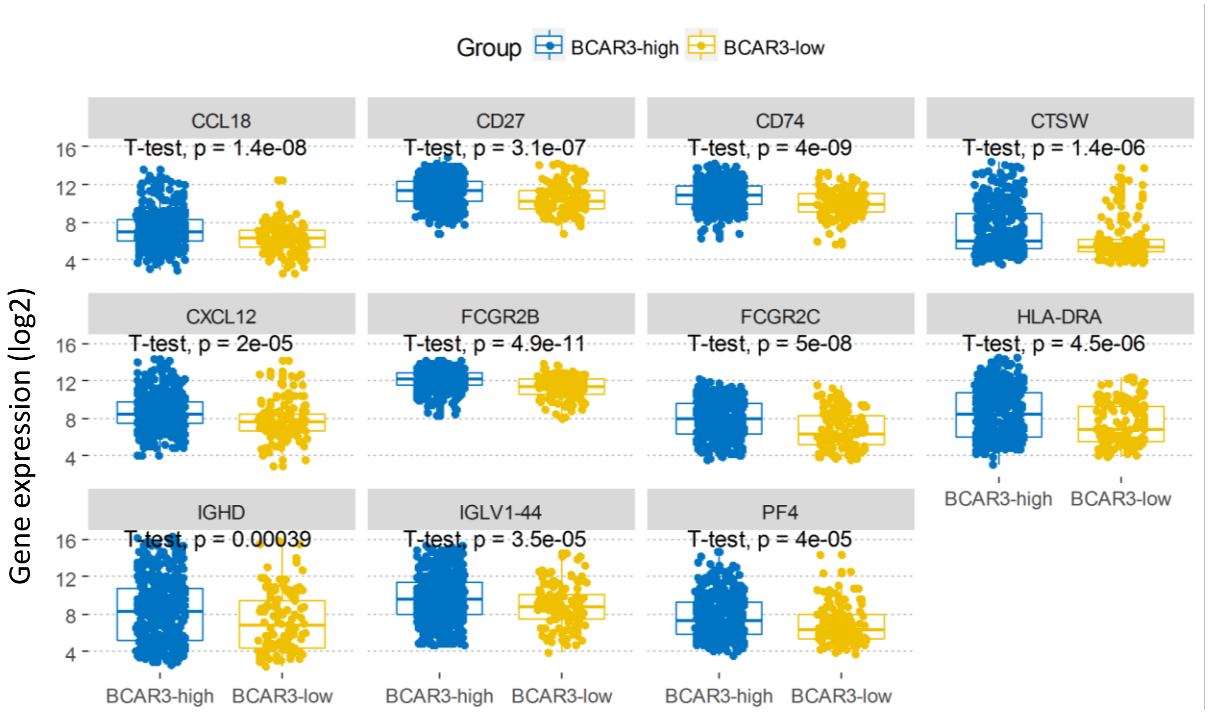

Additional Figure S5

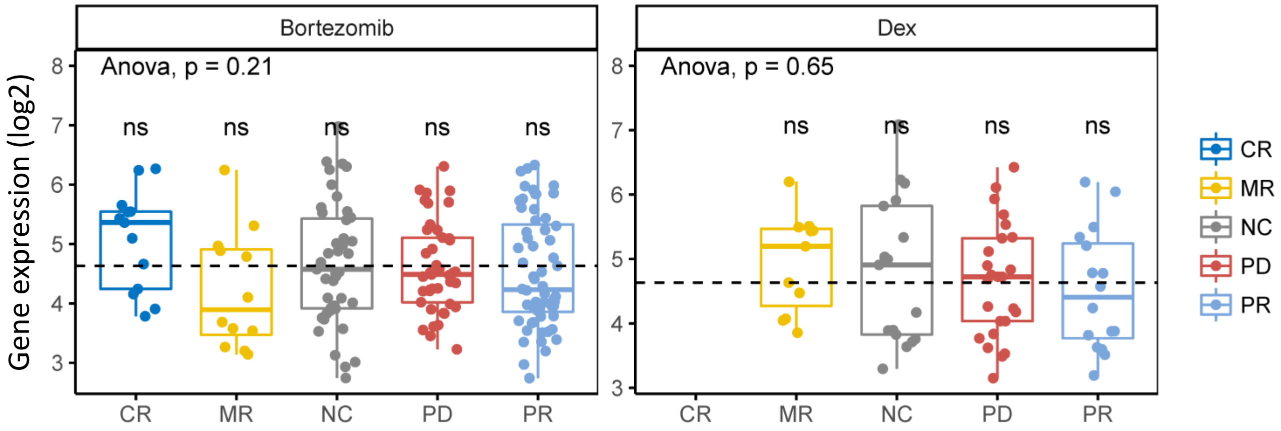

Additional Figure S6

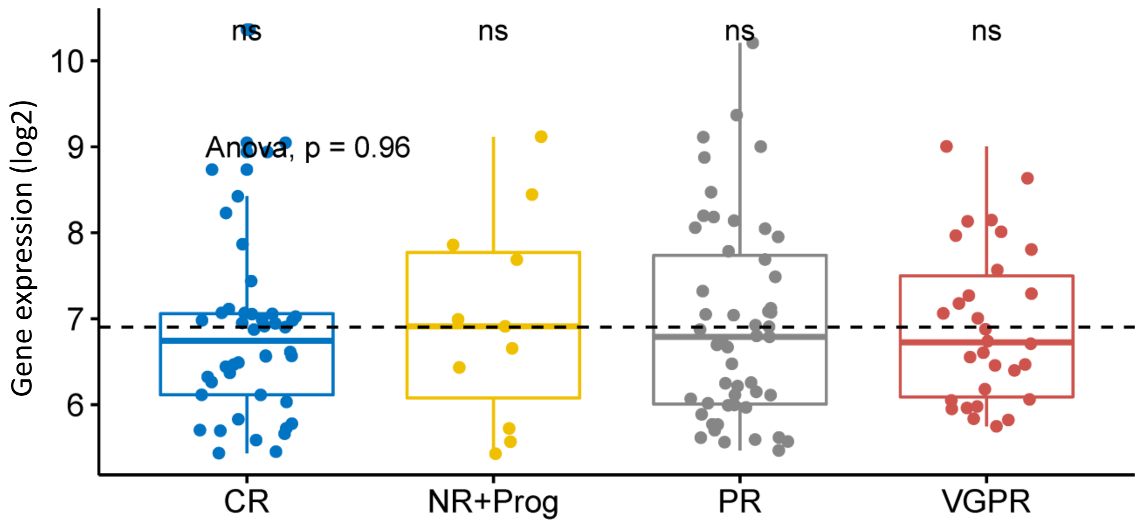

Supplement: Supplementary file 1 — Additional file 1: Figure S1. The expression level of BCAR3 in different molecular subtypes. The X-axis represents the 9 molecular subtypes; the Y-axis represents the gene expression. The dotted line represents the average of BCAR3 gene expression levels of all molecular subtypes, including CD1, CD2, CTA, HY, MF, MS, myeloid, NFKB, PR. P = 7.8E−10, Anova test. Figure S2. The expression level of BCAR3 in different ISS stages and 3 serotypes in MM patients. A, The expression of BCAR3 was compared between different ISS stages in MM patients. The X-axis represents the ISS stages; the Y-axis represents BCAR3 expression level (log2). P = 0.00068, Kruskal–Wallis test. B, The expression of BCAR3 in different ISS stages were compared under different serotype stratification (FLC: Serum free light chain, IgA: Serum immunoglobulin A, IgG: serum immunoglobulin G). Kruskal–Wallis test. Figure S3. Heat map of different expression genes between BCAR3-low and BCAR3-high groups and related enrichment pathways. A, Heat map shows top 12 up-regulated genes and top 12 down-regulated genes. The red represents high expression, the white represents intermediate expression, and the green represents low expression. The foldchange (log2) of different expressed genes is ranked, and the corresponding P-value (− log10) is on the right in the heat map. B, The enrichment pathways for different expression genes. The X-axis represents p-value (− log10) and the Y-axis represents different enriched pathways. Figure S4. The expression levels of 11 different genes in the immune response pathway in the BCAR3-high group and the BCAR3-low group were compared. Unpaired t test, two sided. Figure S5. BCAR3 expression in different therapeutic response to bortezomib and dexamethasone. The left side shows the therapeutic response to bortezomib. The therapeutic response to dexamethasone was shown on the right. The expressions of BCAR3 were compared between complete remission (CR), partial remission (PR), minimal r [file 12967_2018_1728_MOESM1_ESM.pdf]
