# Supplementary material for: Prediction and prognostic significance of BCAR3 expression in patients with multiple myeloma
Source: J Transl Med. 2018 Dec 18;16:363. doi: 10.1186/s12967-018-1728-8 (PMC6299524; doi:10.1186/s12967-018-1728-8)
Supplement: Supplementary file 2 — Additional file 2: Table S1. Multivariate analysis of clinical prognostic parameters in 559 multiple myeloma patients (Cox regression multivariate analysis). Table S2. Baseline patient characteristics according to the expression level of BCAR3. [file 12967_2018_1728_MOESM2_ESM.docx]

Additional Table S1. Multivariate analysis of clinical prognostic parameters in 559 multiple myeloma patients (Cox regression multivariate analysis)

|  |  | 95% CI for HR | |  |
| --- | --- | --- | --- | --- |
|  | HR | Lower | Upper | *P*-value |
| EFS |  |  |  |  |
| B2M (>= 3.5 mg/l) | 1.38 | 1.01 | 1.88 | 4.22E-02 |
| ALB (>= 35 g/l) | 0.85 | 0.60 | 1.20 | 3.57E-01 |
| HGB (>= 100 g/l) | 0.76 | 0.56 | 1.03 | 7.53E-02 |
| MRI (>= 3 focal lesions) | 1.40 | 1.07 | 1.84 | 1.47E-02 |
| BMPC (>= 35%) | 1.39 | 1.01 | 1.92 | 4.56E-02 |
| BCAR3 (>=8.8) | 0.66 | 0.50 | 0.88 | 5.17E-03 |
|  |  |  |  |  |
| OS |  |  |  |  |
| B2M (>= 3.5 mg/l) | 1.59 | 1.09 | 2.33 | 1.74E-02 |
| ALB (>= 35 g/l) | 0.71 | 0.48 | 1.05 | 8.50E-02 |
| HGB (>= 100 g/l) | 0.86 | 0.60 | 1.23 | 3.99E-01 |
| MRI (>= 3 focal lesions) | 1.80 | 1.28 | 2.52 | 7.52E-04 |
| BMPC (>= 35%) | 1.27 | 0.85 | 1.89 | 2.46E-01 |
| BCAR3 (>=8.8) | 0.55 | 0.39 | 0.76 | 3.33E-04 |

ALB, Albumin, g/l; B2M, Beta-2 microglobulin, mg/l; HGB, Haemoglobin, g/l; BMPC, Bone marrow biopsy plasma cells (%); MRI, Number of magnetic resonance imaging (MRI)-defined focal lesions (skull, spine, pelvis); OS, Overall survival time (months), defined from date of registration to the date of death from any cause or censored at the date of last contact. EFS, Event-free survival time (months), defined from date of registration to the occurrence of death from any cause, disease progression or relapse, or censored at the date of last contact; CI, confidence interval; HR, hazard ratio.

Additional Table S2. Baseline patient characteristics according to the expression level of BCAR3.

|  |  | BCAR3-low | BCAR3-high | *P*-value |
| --- | --- | --- | --- | --- |
| n |  | 133 | 426 |  |
| AGE (mean (sd)) |  | 58.06 (9.01) | 56.90 (9.59) | 0.219 |
| SEX (%) | female | 55 (41.4) | 167 (39.2) | 0.733 |
|  | male | 78 (58.6) | 259 (60.8) |  |
| RACE (%) | other | 14 (10.5) | 48 (11.3) | 0.937 |
|  | white | 119 (89.5) | 378 (88.7) |  |
| ISOTYPE (%) | FLC | 15 (12.0) | 69 (16.7) | 0.414 |
|  | IgA | 38 (30.4) | 95 (22.9) |  |
|  | IgD | 1 ( 0.8) | 2 ( 0.5) |  |
|  | IgG | 70 (56.0) | 243 (58.7) |  |
|  | Nonsecretory | 1 ( 0.8) | 5 ( 1.2) |  |
| B2M (mean (sd)) |  | 5.89 (6.83) | 4.37 (4.77) | 0.004 |
| CRP (mean (sd)) |  | 12.21 (20.58) | 11.45 (23.72) | 0.738 |
| CREAT (mean (sd)) |  | 1.40 (1.34) | 1.30 (1.25) | 0.426 |
| LDH (mean (sd)) |  | 185.47 (84.74) | 167.76 (58.33) | 0.007 |
| ALB (mean (sd)) |  | 3.97 (0.64) | 4.07 (0.56) | 0.082 |
| HGB (mean (sd)) |  | 10.97 (1.78) | 11.34 (1.81) | 0.038 |
| ASPC (mean (sd)) |  | 46.79 (24.55) | 41.41 (24.15) | 0.031 |
| BMPC (mean (sd)) |  | 55.63 (24.91) | 43.54 (26.06) | <0.001 |
| MRI (mean (sd)) |  | 14.90 (15.99) | 9.79 (13.83) | 0.001 |

n, number of patients; CRP, CREAT, Creatinine, mg/dl; C-reactive protein, mg/l; ASPC, Aspirate plasma cells (%).
